# Dibromido{(*E*)-2-eth­oxy-6-[3-(methyl­ammonio)propyl­iminometh­yl]phenol­ato}zinc(II)

**DOI:** 10.1107/S1600536808023672

**Published:** 2008-07-31

**Authors:** Xue-Wen Zhu, Xu-Zhao Yang

**Affiliations:** a Key Laboratory of Surface and Interface Science of Henan, School of Materials and Chemical Engineering, Zhengzhou University of Light Industry, Zhengzhou 450002, People’s Republic of China

## Abstract

The title complex, [ZnBr_2_(C_13_H_20_N_2_O_2_)], is a mononuclear zinc(II) compound derived from the zwitterionic form of the Schiff base (*E*)-2-eth­oxy-6-((3-(methyl­amino)propyl­imino)meth­yl)phenol. The Zn^II^ atom is four-coordinated by the imine N and phenolate O atoms of the Schiff base ligand, and by two bromide ions, in a tetra­hedral coordination geometry. Adjacent mol­ecules are linked through inter­molecular N—H⋯O hydrogen bonds, forming chains running along the *b* axis.

## Related literature

For background to the chemistry of the Schiff base complexes, see: Ali *et al.* (2008 ▶); Biswas *et al.* (2008 ▶); Chen *et al.* (2008 ▶); Darensbourg & Frantz (2007 ▶); Habibi *et al.* (2007 ▶); Kawamoto *et al.* (2008 ▶); Lipscomb & Sträter (1996 ▶); Tomat *et al.* (2007 ▶); Wu *et al.* (2008 ▶); Yuan *et al.* (2007 ▶). For related structures, see: Qiu (2006 ▶); Wei *et al.* (2007 ▶); Zhu *et al.* (2007 ▶).
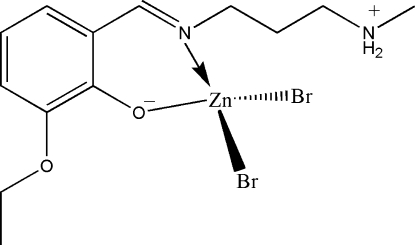


## Experimental

### 

#### Crystal data

[ZnBr_2_(C_13_H_20_N_2_O_2_)]
*M* *_r_* = 461.50
Monoclinic, 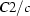

*a* = 17.884 (3) Å
*b* = 14.374 (2) Å
*c* = 14.992 (2) Å
β = 114.482 (3)°
*V* = 3507.4 (9) Å^3^
*Z* = 8
Mo *K*α radiation
μ = 5.96 mm^−1^
*T* = 298 (2) K
0.23 × 0.21 × 0.21 mm

#### Data collection

Bruker APEXII CCD area-detector diffractometer
Absorption correction: multi-scan (*SADABS*; Sheldrick, 2004 ▶) *T*_min_ = 0.341, *T*_max_ = 0.367 (expected range = 0.265–0.286)
14138 measured reflections
3787 independent reflections
1974 reflections with *I* > 2σ(*I*)
*R*_int_ = 0.068

#### Refinement

*R*[*F* ^2^ > 2σ(*F* ^2^)] = 0.067
*wR*(*F* ^2^) = 0.222
*S* = 1.00
3787 reflections
183 parameters
H-atom parameters constrained
Δρ_max_ = 1.12 e Å^−3^
Δρ_min_ = −0.90 e Å^−3^

### 

Data collection: *APEX2* (Bruker, 2004 ▶); cell refinement: *SAINT* (Bruker, 2004 ▶); data reduction: *SAINT*; program(s) used to solve structure: *SHELXS97* (Sheldrick, 2008 ▶); program(s) used to refine structure: *SHELXL97* (Sheldrick, 2008 ▶); molecular graphics: *SHELXTL* (Sheldrick, 2008 ▶); software used to prepare material for publication: *SHELXTL*.

## Supplementary Material

Crystal structure: contains datablocks global, I. DOI: 10.1107/S1600536808023672/sj2525sup1.cif

Structure factors: contains datablocks I. DOI: 10.1107/S1600536808023672/sj2525Isup2.hkl

Additional supplementary materials:  crystallographic information; 3D view; checkCIF report

## Figures and Tables

**Table:** 

Zn1—O1	1.958 (5)
Zn1—N1	2.014 (6)
Zn1—Br1	2.3429 (16)
Zn1—Br2	2.4046 (18)

**Table:** 

O1—Zn1—N1	95.3 (2)
O1—Zn1—Br1	115.26 (16)
N1—Zn1—Br1	113.83 (19)
O1—Zn1—Br2	113.02 (17)
N1—Zn1—Br2	113.09 (19)
Br1—Zn1—Br2	106.38 (6)

**Table 2:** Hydrogen-bond geometry (Å, °)

*D*—H⋯*A*	*D*—H	H⋯*A*	*D*⋯*A*	*D*—H⋯*A*
N2—H2*B*⋯O1^i^	0.90	1.84	2.697 (8)	158
N2—H2*B*⋯O2^i^	0.90	2.40	3.005 (8)	124

Symmetry code: (i) 
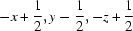
.

**Fig. 1.**
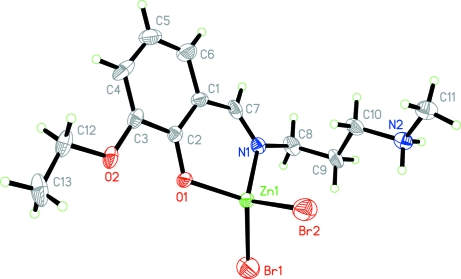
The molecular structure of (I) with ellipsoids drawn at the 30% probability level.

**Fig. 2.**
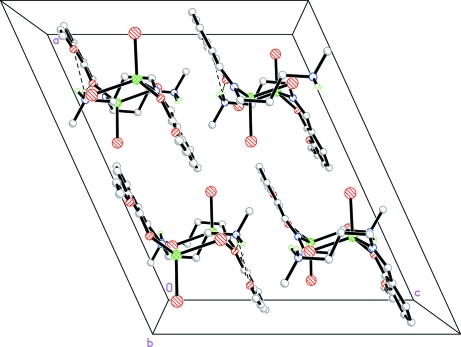
The crystal packing of (I), viewed along the c axis.

## supplementary crystallographic information

### Comment

Schiff bases have been widely used as versatile ligands in coordination chemistry (Biswas *et al.*, 2008; Wu *et al.*, 2008; Kawamoto *et al.*, 2008; Ali *et al.*, 2008; Habibi *et al.*, 2007), and their metal complexes are of great interest in many fields (Chen *et al.*, 2008; Yuan *et al.*, 2007; Tomat *et al.*, 2007; Darensbourg & Frantz, 2007). Zinc(II) is an important element in biological systems, functions as the active site of hydrolytic enzymes, such as carboxypeptidase and carbonic anhydrase where it is in a hard-donor coordination environment of nitrogen and oxygen ligands (Lipscomb & Sträter, 1996). In this paper, a new zinc(II) complex, (I), Fig. 1, with the Schiff base ligand 2-ethoxy-6-[(3-methylaminopropylimino)methyl]phenol has been synthesized and structurally characterized.

The Zn^II^ atom in (I) is four-coordinated by the imine N and phenolate O atoms of the zwitterionic form of the Schiff base ligand and by two Br^-^ ions in a tetrahedral coordination geometry. The coordinate bond lengths (Table 1) are typical and comparable to the corresponding values observed in other similar zinc(II) Schiff base complexes (Zhu *et al.*, 2007; Wei *et al.*, 2007; Qiu, 2006).

In the crystal structure, adjacent molecules are linked through intermolecular N–H···O hydrogen bonds (Table 2), forming chains running along the *b* axis (Fig. 2).

### Experimental

The Schiff base compound was prepared by the condensation of equimolar amounts of 3-ethoxysalicylaldehyde with *N*-methylpropane-1,3-diamine in a methanol solution. The complex was prepared by the following method. To an anhydrous methanol solution (5 ml) of ZnBr_2_ (22.5 mg, 0.1 mmol) was added a methanol solution (10 ml) of the Schiff base compound (23.6 mg, 0.1 mmol) with stirring. The mixture was stirred for 30 min at room temperature and filtered. Upon keeping the filtrate in air for a few days, colorless block-shaped crystals were formed at the bottom of the vessel on slow evaporation of the solvent.

### Refinement

All H atoms were placed in geometrically idealized positions and constrained to ride on their parent atoms, with C–H distances in the range 0.93–0.97 Å, N–H distances of 0.90 Å, and with *U*_iso_(H) = 1.2*U*_eq_(C,*N*) and 1.5*U*_eq_(methyl C). The structure contains a solvent accessible void of 58 Å^3^, which might accommodate a disordered methanol solvent molecule.

### Crystal data

**Table:**

[ZnBr_2_(C_13_H_20_N_2_O_2_)]	*F*_000_ = 1824
*M**_r_* = 461.50	*D*_x_ = 1.748 Mg m^−^^3^
Monoclinic, *C*2/*c*	Mo *K*α radiation λ = 0.71073 Å
Hall symbol: -C 2yc	Cell parameters from 1842 reflections
*a* = 17.884 (3) Å	θ = 2.4–24.1º
*b* = 14.374 (2) Å	µ = 5.96 mm^−^^1^
*c* = 14.992 (2) Å	*T* = 298 (2) K
β = 114.482 (3)º	Block, colorless
*V* = 3507.4 (9) Å^3^	0.23 × 0.21 × 0.21 mm
*Z* = 8	

### Data collection

**Table:**

Bruker APEXII CCD area-detector diffractometer	3787 independent reflections
Radiation source: fine-focus sealed tube	1974 reflections with *I* > 2σ(*I*)
Monochromator: graphite	*R*_int_ = 0.068
*T* = 298(2) K	θ_max_ = 27.0º
ω scans	θ_min_ = 1.9º
Absorption correction: multi-scan(SADABS; Sheldrick, 2004)	*h* = −22→22
*T*_min_ = 0.341, *T*_max_ = 0.368	*k* = −18→18
14138 measured reflections	*l* = −18→18

### Refinement

**Table:**

Refinement on *F*^2^	Secondary atom site location: difference Fourier map
Least-squares matrix: full	Hydrogen site location: inferred from neighbouring sites
*R*[*F*^2^ > 2σ(*F*^2^)] = 0.067	H-atom parameters constrained
*wR*(*F*^2^) = 0.222	*w* = 1/[σ^2^(*F*_o_^2^) + (0.1251*P*)^2^] where *P* = (*F*_o_^2^ + 2*F*_c_^2^)/3
*S* = 1.00	(Δ/σ)_max_ = 0.001
3787 reflections	Δρ_max_ = 1.12 e Å^−^^3^
183 parameters	Δρ_min_ = −0.90 e Å^−^^3^
Primary atom site location: structure-invariant direct methods	Extinction correction: none

### Special details

**Table:**

Geometry. All e.s.d.'s (except the e.s.d. in the dihedral angle between two l.s. planes) are estimated using the full covariance matrix. The cell e.s.d.'s are taken into account individually in the estimation of e.s.d.'s in distances, angles and torsion angles; correlations between e.s.d.'s in cell parameters are only used when they are defined by crystal symmetry. An approximate (isotropic) treatment of cell e.s.d.'s is used for estimating e.s.d.'s involving l.s. planes.
Refinement. Refinement of *F*^2^ against ALL reflections. The weighted *R*-factor *wR* and goodness of fit *S* are based on *F*^2^, conventional *R*-factors *R* are based on *F*, with *F* set to zero for negative *F*^2^. The threshold expression of *F*^2^ > σ(*F*^2^) is used only for calculating *R*-factors(gt) *etc*. and is not relevant to the choice of reflections for refinement. *R*-factors based on *F*^2^ are statistically about twice as large as those based on *F*, and *R*- factors based on ALL data will be even larger.

### Fractional atomic coordinates and isotropic or equivalent isotropic displacement parameters (Å^2^)

**Table:**

	*x*	*y*	*z*	*U*_iso_*/*U*_eq_	
Zn1	0.27393 (5)	0.33839 (6)	0.31911 (7)	0.0412 (3)	
Br1	0.41500 (7)	0.37049 (9)	0.38443 (10)	0.0911 (5)	
Br2	0.22492 (8)	0.35250 (9)	0.14417 (9)	0.0842 (4)	
O1	0.2106 (3)	0.4129 (3)	0.3728 (4)	0.0457 (13)	
O2	0.1034 (3)	0.5348 (4)	0.3750 (4)	0.0524 (15)	
N1	0.2463 (4)	0.2150 (4)	0.3615 (5)	0.0435 (15)	
N2	0.2234 (4)	0.0849 (4)	0.0895 (5)	0.0506 (17)	
H2A	0.2508	0.1333	0.0793	0.061*	
H2B	0.2536	0.0335	0.0945	0.061*	
C1	0.1272 (5)	0.2825 (6)	0.3792 (6)	0.049 (2)	
C2	0.1426 (4)	0.3790 (5)	0.3754 (5)	0.0394 (17)	
C3	0.0835 (5)	0.4402 (6)	0.3797 (5)	0.047 (2)	
C4	0.0121 (5)	0.4091 (8)	0.3825 (6)	0.065 (3)	
H4	−0.0272	0.4517	0.3819	0.078*	
C5	−0.0022 (6)	0.3135 (8)	0.3860 (7)	0.074 (3)	
H5	−0.0509	0.2930	0.3879	0.089*	
C6	0.0554 (6)	0.2503 (8)	0.3869 (7)	0.068 (3)	
H6	0.0473	0.1870	0.3924	0.082*	
C7	0.1826 (6)	0.2104 (5)	0.3813 (6)	0.055 (2)	
H7	0.1713	0.1520	0.3995	0.066*	
C8	0.2915 (6)	0.1308 (6)	0.3638 (7)	0.057 (2)	
H8A	0.2641	0.0783	0.3781	0.069*	
H8B	0.3462	0.1354	0.4162	0.069*	
C9	0.2982 (5)	0.1139 (6)	0.2700 (6)	0.055 (2)	
H9A	0.3320	0.0593	0.2767	0.067*	
H9B	0.3253	0.1665	0.2557	0.067*	
C10	0.2170 (5)	0.0998 (6)	0.1873 (7)	0.057 (2)	
H10A	0.1828	0.1536	0.1818	0.068*	
H10B	0.1906	0.0461	0.2009	0.068*	
C11	0.1458 (5)	0.0750 (7)	0.0041 (7)	0.065 (3)	
H11A	0.1211	0.0166	0.0073	0.097*	
H11B	0.1554	0.0771	−0.0543	0.097*	
H11C	0.1097	0.1249	0.0029	0.097*	
C12	0.0459 (6)	0.6012 (7)	0.3834 (7)	0.065 (3)	
H12A	0.0413	0.5924	0.4450	0.079*	
H12B	−0.0079	0.5922	0.3304	0.079*	
C13	0.0754 (6)	0.6948 (8)	0.3788 (8)	0.084 (4)	
H13A	0.1295	0.7025	0.4299	0.127*	
H13B	0.0389	0.7395	0.3872	0.127*	
H13C	0.0771	0.7041	0.3163	0.127*	

### Atomic displacement parameters (Å^2^)

**Table:**

	*U*^11^	*U*^22^	*U*^33^	*U*^12^	*U*^13^	*U*^23^
Zn1	0.0429 (5)	0.0361 (5)	0.0501 (6)	0.0014 (4)	0.0248 (4)	−0.0029 (4)
Br1	0.0580 (7)	0.0846 (8)	0.1261 (11)	0.0068 (5)	0.0335 (7)	−0.0125 (7)
Br2	0.0864 (8)	0.0953 (9)	0.0692 (8)	−0.0150 (6)	0.0305 (6)	0.0043 (6)
O1	0.042 (3)	0.036 (3)	0.071 (4)	0.001 (2)	0.034 (3)	−0.006 (3)
O2	0.046 (3)	0.061 (4)	0.051 (4)	0.019 (3)	0.021 (3)	−0.007 (3)
N1	0.049 (4)	0.038 (4)	0.042 (4)	0.000 (3)	0.017 (3)	−0.001 (3)
N2	0.050 (4)	0.035 (4)	0.061 (5)	0.005 (3)	0.018 (4)	0.006 (3)
C1	0.046 (5)	0.064 (6)	0.041 (5)	−0.017 (4)	0.022 (4)	−0.006 (4)
C2	0.040 (4)	0.046 (4)	0.030 (4)	0.000 (3)	0.012 (3)	−0.002 (3)
C3	0.044 (5)	0.067 (6)	0.032 (5)	0.012 (4)	0.017 (4)	0.001 (4)
C4	0.039 (5)	0.113 (9)	0.050 (6)	0.013 (5)	0.026 (4)	0.012 (5)
C5	0.057 (6)	0.103 (9)	0.069 (7)	−0.030 (6)	0.033 (5)	−0.011 (6)
C6	0.066 (6)	0.085 (7)	0.066 (7)	−0.013 (6)	0.039 (6)	−0.002 (6)
C7	0.084 (7)	0.029 (4)	0.053 (6)	−0.005 (4)	0.030 (5)	−0.006 (4)
C8	0.078 (6)	0.030 (4)	0.057 (6)	0.009 (4)	0.021 (5)	0.008 (4)
C9	0.051 (5)	0.042 (5)	0.061 (6)	0.002 (4)	0.012 (4)	−0.019 (4)
C10	0.042 (5)	0.043 (5)	0.078 (7)	−0.001 (4)	0.018 (5)	−0.014 (5)
C11	0.059 (6)	0.065 (6)	0.064 (6)	−0.005 (5)	0.019 (5)	0.010 (5)
C12	0.070 (6)	0.078 (7)	0.055 (6)	0.038 (5)	0.032 (5)	0.006 (5)
C13	0.084 (7)	0.074 (7)	0.071 (7)	0.038 (6)	0.007 (6)	−0.024 (6)

### Geometric parameters (Å, °)

**Table:**

Zn1—O1	1.958 (5)	C5—H5	0.9300
Zn1—N1	2.014 (6)	C6—H6	0.9300
Zn1—Br1	2.3429 (16)	C7—H7	0.9300
Zn1—Br2	2.4046 (18)	C8—C9	1.480 (12)
O1—C2	1.325 (8)	C8—H8A	0.9700
O2—C3	1.414 (11)	C8—H8B	0.9700
O2—C12	1.446 (9)	C9—C10	1.481 (11)
N1—C7	1.293 (11)	C9—H9A	0.9700
N1—C8	1.447 (10)	C9—H9B	0.9700
N2—C11	1.453 (10)	C10—H10A	0.9700
N2—C10	1.532 (11)	C10—H10B	0.9700
N2—H2A	0.9000	C11—H11A	0.9600
N2—H2B	0.9000	C11—H11B	0.9600
C1—C6	1.414 (12)	C11—H11C	0.9600
C1—C2	1.420 (11)	C12—C13	1.456 (15)
C1—C7	1.423 (12)	C12—H12A	0.9700
C2—C3	1.396 (11)	C12—H12B	0.9700
C3—C4	1.370 (12)	C13—H13A	0.9600
C4—C5	1.403 (15)	C13—H13B	0.9600
C4—H4	0.9300	C13—H13C	0.9600
C5—C6	1.369 (14)		
			
O1—Zn1—N1	95.3 (2)	C1—C7—H7	115.5
O1—Zn1—Br1	115.26 (16)	N1—C8—C9	112.2 (7)
N1—Zn1—Br1	113.83 (19)	N1—C8—H8A	109.2
O1—Zn1—Br2	113.02 (17)	C9—C8—H8A	109.2
N1—Zn1—Br2	113.09 (19)	N1—C8—H8B	109.2
Br1—Zn1—Br2	106.38 (6)	C9—C8—H8B	109.2
C2—O1—Zn1	120.3 (4)	H8A—C8—H8B	107.9
C3—O2—C12	115.4 (7)	C8—C9—C10	112.4 (8)
C7—N1—C8	119.3 (7)	C8—C9—H9A	109.1
C7—N1—Zn1	118.1 (5)	C10—C9—H9A	109.1
C8—N1—Zn1	122.5 (6)	C8—C9—H9B	109.1
C11—N2—C10	115.7 (7)	C10—C9—H9B	109.1
C11—N2—H2A	108.4	H9A—C9—H9B	107.9
C10—N2—H2A	108.4	C9—C10—N2	112.7 (7)
C11—N2—H2B	108.4	C9—C10—H10A	109.1
C10—N2—H2B	108.4	N2—C10—H10A	109.1
H2A—N2—H2B	107.4	C9—C10—H10B	109.1
C6—C1—C2	121.3 (8)	N2—C10—H10B	109.1
C6—C1—C7	114.0 (8)	H10A—C10—H10B	107.8
C2—C1—C7	124.6 (7)	N2—C11—H11A	109.5
O1—C2—C3	119.4 (7)	N2—C11—H11B	109.5
O1—C2—C1	123.7 (7)	H11A—C11—H11B	109.5
C3—C2—C1	116.8 (7)	N2—C11—H11C	109.5
C4—C3—C2	121.9 (9)	H11A—C11—H11C	109.5
C4—C3—O2	124.8 (8)	H11B—C11—H11C	109.5
C2—C3—O2	113.1 (7)	O2—C12—C13	108.8 (8)
C3—C4—C5	120.5 (9)	O2—C12—H12A	109.9
C3—C4—H4	119.8	C13—C12—H12A	109.9
C5—C4—H4	119.8	O2—C12—H12B	109.9
C6—C5—C4	120.1 (9)	C13—C12—H12B	109.9
C6—C5—H5	119.9	H12A—C12—H12B	108.3
C4—C5—H5	119.9	C12—C13—H13A	109.5
C5—C6—C1	119.2 (9)	C12—C13—H13B	109.5
C5—C6—H6	120.4	H13A—C13—H13B	109.5
C1—C6—H6	120.4	C12—C13—H13C	109.5
N1—C7—C1	129.0 (8)	H13A—C13—H13C	109.5
N1—C7—H7	115.5	H13B—C13—H13C	109.5

### Hydrogen-bond geometry (Å, °)

**Table:**

*D*—H···*A*	*D*—H	H···*A*	*D*···*A*	*D*—H···*A*
N2—H2B···O1^i^	0.90	1.84	2.697 (8)	158
N2—H2B···O2^i^	0.90	2.40	3.005 (8)	124

Symmetry codes: (i) −*x*+1/2, *y*−1/2, −*z*+1/2.
